# The potassium channel *FaTPK1* plays a critical role in fruit quality formation in strawberry (*Fragaria* × *ananassa*)

**DOI:** 10.1111/pbi.12824

**Published:** 2017-10-12

**Authors:** Shufang Wang, Miaoyu Song, Jiaxuan Guo, Yun Huang, Fangfang Zhang, Cheng Xu, Yinghui Xiao, Lusheng Zhang

**Affiliations:** ^1^ College of Horticulture China Agricultural University Beijing China; ^2^ Department of resources and environment Beijing University of Agriculture Beijing China

**Keywords:** Potassium channel gene *FaTPK1*, strawberry fruit ripening, RNA interference and overexpression, Green fluorescent protein subcellular localization, protein expression

## Abstract

Potassium (K^+^), an abundant cation in plant cells, is important in fruit development and plant resistance. However, how cellular K^+^ is directed by potassium channels in fruit development and quality formation of strawberry (*Fragaria* × *ananassa*) is not yet fully clear. Here, a two‐pore K^+^ (TPK) channel gene in strawberry, *FaTPK1*, was cloned using reverse transcription–PCR. A green fluorescent protein subcellular localization analysis showed that *FaTPK1* localized in the vacuole membrane. A transcription analysis indicated that the mRNA expression level of *FaTPK1* increased rapidly and was maintained at a high level in ripened fruit, which was coupled with the fruit's red colour development, suggesting that *FaTPK1* is related to fruit quality formation. The down‐ and up‐regulation of the *FaTPK1* mRNA expression levels using RNA interference and overexpression, respectively, inhibited and promoted fruit ripening, respectively, as demonstrated by consistent changes in firmness and the contents of soluble sugars, anthocyanin and abscisic acid, as well as the transcript levels of ripening‐regulated genes *PG1* (polygalacturonase), *GAL6* (beta‐galactosidase), *XYL2* (D‐xylulose reductase), *SUT1* (sucrose transporter), *CHS* (chalcone synthase) and *CHI* (chalcone flavanone isomerase). Additionally, the regulatory changes influenced fruit resistance to *Botrytis cinerea*. An isothermal calorimetry analysis showed that the *Escherichia coli*‐expressed *FaTPK1* recombinant protein could bind K^+^ with a binding constant of 2.1 × 10^–3^ m ^−1^ and a dissociation constant of 476 μm. Thus, the strawberry *TPK1* is a ubiquitously expressed, tonoplast‐localized two‐pore potassium channel that plays important roles in fruit ripening and quality formation.

## Introduction

Potassium (K^+^), an important and abundant cation in plants, is not only involved in anion neutralization, pH homeostasis, and mediating membrane electrical potential and cell osmotic pressure but also takes part in protein synthesis, cell metabolism and photosynthesis. Thus, a large quantity of K^+^ is essential and inevitable for plant growth and development, processes in which the cellular vacuoles play critical roles (Latz *et al*., 2007; Nieves‐Cordones *et al*., 2016; Sharma *et al*., 2013). Although K^+^ also influences fruit yield and quality in many crops, such as grapevine, maize, wheat, soya bean and cotton (Conde *et al*., 2006; Pettigrew, 2008), how K^+^ is directed by K^+^ channels in the vacuoles of fleshy fruits is yet unknown.

In plants, K^+^ uptake and fluxes are mediated by several families of transporters and channels, including Shaker, two‐pore K^+^ (TPK) channels and K^+^ inward rectifier‐like channels (Lebaudy *et al*., 2007; Sharma *et al*., 2013). In plants, TPK proteins, as vacuolar K^+^ channels with two‐pore domains, are localized to the vacuolar membrane and play pivotal roles in maintaining K^+^ homeostasis (Hedrich, 2012; Latz *et al*., 2007; Lebaudy *et al*., 2007). In the model plant *Arabidopsis*, there are six members of the K^+^ channels, five *TPK*s (*TPK1*–*TPK5*) and a single K^+^ inward rectifier‐like channel, that are located on the vacuolar membrane, except for *TPK4*, which localizes to the plasma membrane (Voelker *et al*., 2010). *AtTPK1* mediates intracellular K^+^ homeostasis between cytoplasmic and vacuolar compartments, and is a voltage‐independent, Ca^2+^/pH‐activated K^+^ channel that functions in germination, seedling growth, stomatal closure and intracellular osmosensing through interactions with 14‐3‐3 proteins in *Arabidopsis* (Enyedi and Czirjak, 2010; Gobert *et al*., 2007; Latz *et al*., 2007; Maathuis, 2011). In contrast, the rice genome only encodes two ubiquitously expressed *TPK* isoforms, *TPKa*, which localizes predominantly to the large lytic vacuole, and *TPKb*, which localizes primarily to the small vacuoles (Isayenkov *et al*., 2011). *TPKb* can alter the K^+^ status of small vacuoles and is important for cellular K^+^ homeostasis in response to stress tolerance (Ahmad *et al*., 2016). In *Nicotiana tabacum*,* NtTPK1* targets the tonoplasts in tobacco cells, exhibiting a strong selectivity for K^+^ over Na^+^, and a higher Ca^2+^ concentration or lower pH markedly increases *NtTPK1*‐mediated K^+^ currents (Hamamoto *et al*., 2008). The vacuolar TPK channels regulated by Ca^2+^, 14‐3‐3 proteins and cytosolic pH, have been characterized only in a few plants.

K^+^ regulates in stomata opening in the guard cells (Allen and Sanders, 1995). The tonoplast *AtTPK1* regulates stomatal closure using K^+^ released from vacuoles (Gobert *et al*., 2007). The accumulation of K^+^ and sugars results in stomatal conductance with increasing water uptake (Shimazaki *et al*., 2007 and Talbott and Zeiger, 1998). The vacuolar K^+^ channel *AtTPK1* plays a main role in ABA‐dependent stomatal closure (Gobert *et al*., 2007).

An osmotic‐driven enlargement of the vacuole promotes fleshy fruit development, which involves in cell division, cell expansion and fruit ripening (Ho, 1996). Thus, fruit quality is dependent on the stored compounds in the vacuole, including pigments, sugars, organic acids and other secondary metabolites (Martinoia *et al*., 2007). Because the TPK channels serve as vacuolar osmosensors (Maathuis, 2011), we postulated that they may play an important role in the regulation of fruit development, ripening and quality formation. To date, although K^+^ is important in fleshy fruit quality formation, to our knowledge, only the grapevine *KAT*‐type K^+^ channels (Pratelli *et al*., 2002) and a role of *FaKAT1* (an ABA‐induced K^+^ channel gene) in strawberry fruit ripening have been reported (Song *et al*., 2017). The *AtKAT1* is a downstream component in ABA signalling through an ABA‐activated SnRK2.6 protein kinase (Sato *et al*., 2009). Because of the vital role of the vacuole in fleshy fruit development and quality formation, the vacuole membrane‐localized *TPK*‐type K^+^ channels need to be identified in fleshy fruits.

The fruit of strawberry (*Fragaria ananassa*) is an ideal model plant in the study of fruit development, especially the ripening of nonclimacteric fruit (Given *et al*., 1988; Li *et al*., 2011). To explore the roles of TPKs in fruit ripening and quality formation, a strawberry *TPK* gene, *FaTPK1*, was identified by RNA‐sequencing and was cloned by reverse transcription‐PCR (RT–PCR); its gene functions and protein attributes were then studied using physiological, molecular and biochemical analysis, including HPLC, virus‐induced gene silencing (VIGS), intron‐containing hairpin RNA (ihpRNA), prokaryotic expression and isothermal calorimetry (iTC). Our results demonstrated that *FaTPK1* is a tonoplast‐localized, fruit quality‐regulating K^+^ channel.

## Results

### Changes of exterior morphology and physiological parameters in developmental strawberry fruit

**Figure 1:**
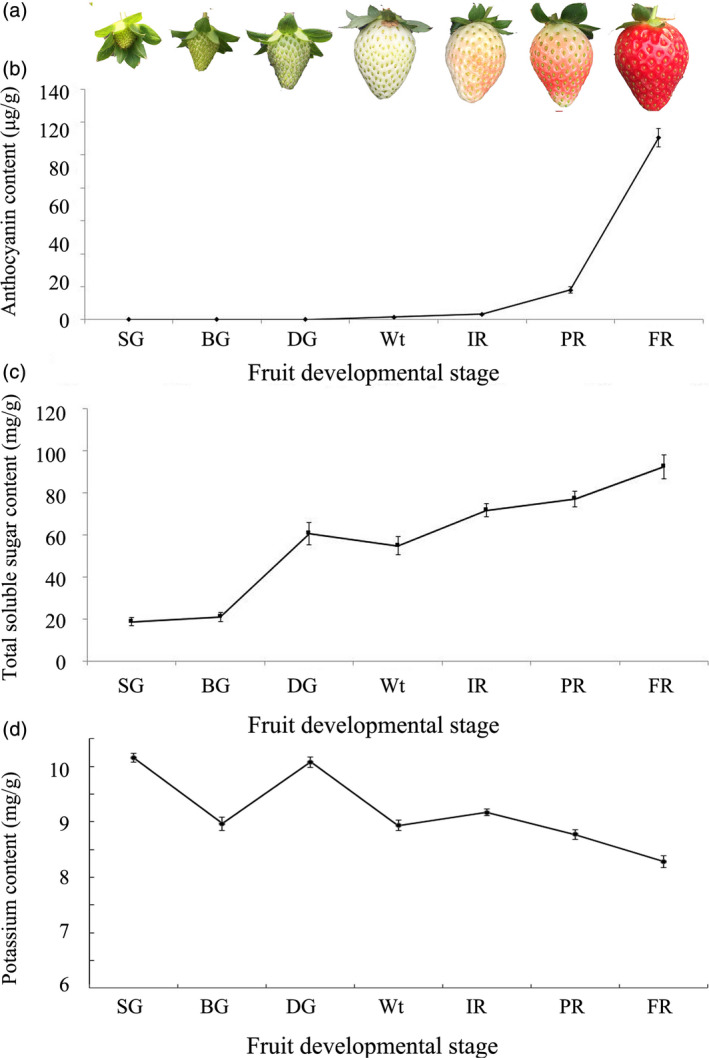
Physiological variations in developmental strawberry fruit. Developmental processes were divided into SG, BG, DG, Wt, IR, PR and FR, and included changes in the (a), anthocyanin content (b), soluble sugar content (c) and potassium content (d). Error bars represent SD (*n* = 3).

Observed morphological processes of ‘Sweet charlie’ strawberry fruit (Chai *et al*., 2011; Jia *et al*., 2011) have been divided into seven developmental stages: small green (SG), big green (BG), de‐green (DG), white (Wt), initial red (IR), partially red (PR) and fully red (FR) (Figure 1a). Distinct physiological changes also occur in the developmental receptacles, with the anthocyanin contents sharply increasing (Figure 1b), and the total soluble sugar contents rapidly and continually increasing after the Wt stage (Figure 1c). The K^+^ contents, on the whole, showed a decreasing and oscillatory trend during the strawberry fruit development (Figure 1d).

### Transcriptome analysis and cloning of *FaTPK1*

**Figure 2:**
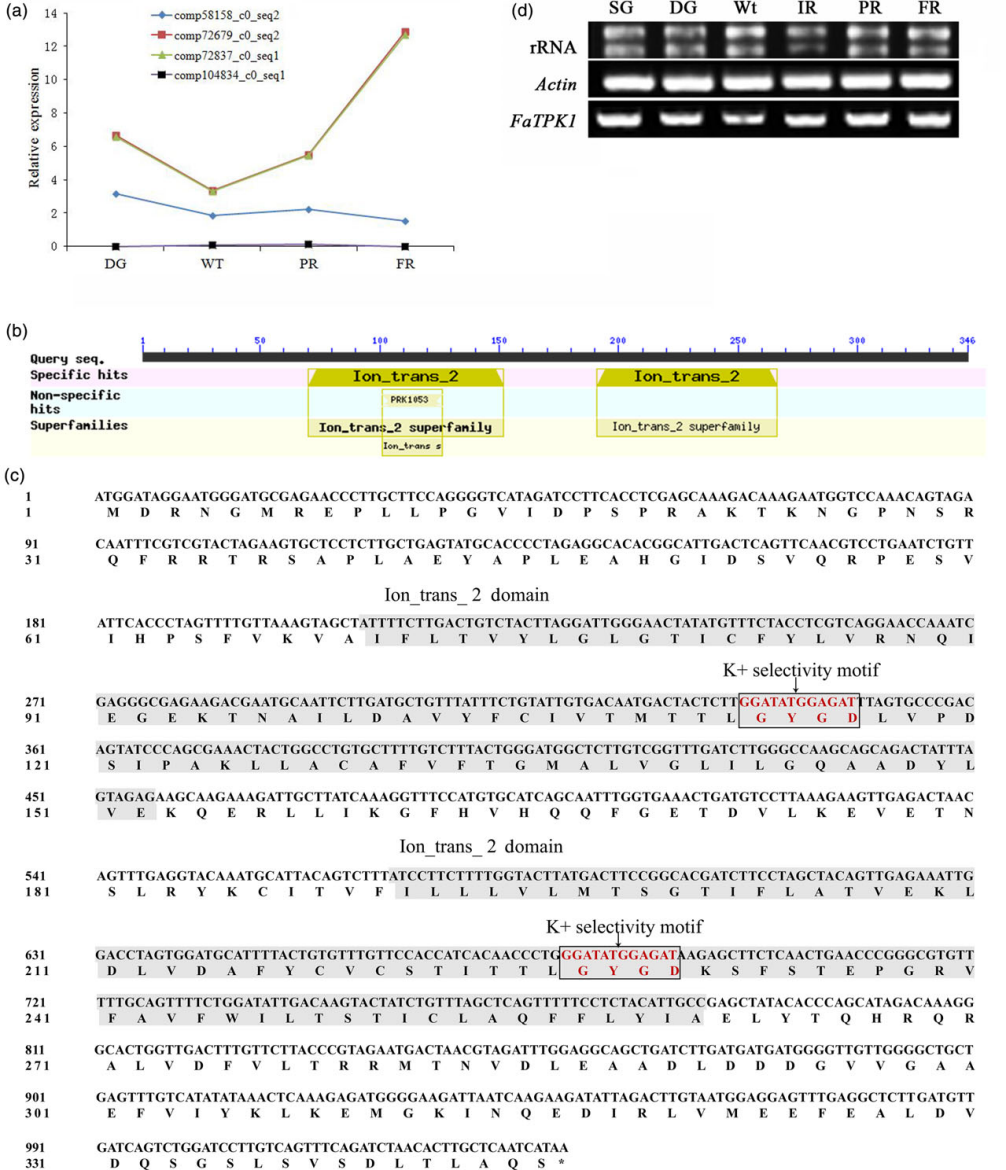
Two‐pore potassium (TPK) channel gene transcriptome data of strawberry, the characterization of the *FaTPK1* gene sequence and its semi‐quantitative detection. (a) Four *TPK TPK*‐like homologues were detected and divided into three groups based on log2 gene expression levels and the four fruit stages, DG, Wt, IR and PR, that occur near the onset of strawberry fruit ripening. The sequence alignment in NCBI found that the first group had two complementary hits, comp72679_c0_seq2 and comp72837_c0_seq1, that aligned with the *Fragaria vesca* TPK channel 1‐like (LOC101294428) sequence, and was named *FaTPK1*; the second group, comp58158_c0_seq2 aligns with *F. vesca* TPK channel 1‐like (LOC101294725); the last group, comp104834_c0_seq1 aligns with *F. vesca* TPK channel 3‐like (LOC101307905). (b) The conserved‐domain regions were determined for the FaTPK1 protein using a 364‐amino acid polypeptide BLAST algorithm‐based search of the NCBI database (http://blast.ncbi.nlm.nih.gov/Blast.cgi). (c) FaTPK1 protein has two ion_trans_2 domains, each pore containing a GYGD K^+^ selectivity motif. (d) Relative gene expression of *FaTPK1* in six developmental processes of strawberry fruits.

Based on transcriptome data (Zhao *et al*., 2017), RNA‐seq (RNA‐sequencing) technology has been performed using the four‐stage (LG, Wt, IR and PR) fruit around the onset of the strawberry fruit. At log2 gene expression levels, four *TPK*‐like homologues were detected and divided into three groups, in which the highest expressing contigs (comp72679_c0_seq2 and comp72837_c0_seq1) were coupled with the fruit's red colouring, and these were used as query in a BLAST algorithm‐based search of the NCBI databases (https://blast.ncbi.nlm.nih.gov/Blast.cgi). They matched the TPK channel 1‐like protein (LOC101294428) in *Fragaria vesca*, and this was named *FaTPK1* (Figure 2a).

To clone the *FaTPK1* gene, the RNA‐seq sequences were used for a query in a BLAST algorithm‐based search of the NCBI databases and a high homologous sequence (GenBank NO. XM_004291514) was identified. Based on the cDNA length, including the coding sequence of *FaTPK1*, the full‐length *FaTPK1* gene was cloned using RT–PCR. A 1,041‐bp sequence was gained and encoded a deduced 346 amino acids. The identity between *FaTPK1* and *FvTPK1* (GenBank NO. XM_004291514.2) is 99.42% (Figure S1), and the amino acid sequence similarity level is the same. A search for both conserved domains using the NCBI website (http://www.ncbi.nlm.nih.gov/structure/cdd/wrpsb.cgi) and transmembrane domains using the website ExPASy (http://www.expasy.org/proteomics) revealed that *FaTPK1* is a transmembrane protein with two Ion_trans_2 conserved domains, which include a GYGD K^+^ selectivity motif (Figure 2b,c).

To further confirm transcripts of the gene in developing fruit, SqRT–PCR was used to investigate the six stages fruit. A similar expression trend for this gene was detected around onset of strawberry fruit ripening (Figure 2d), suggesting that the ripening of strawberry fruit was required for a certain stable expression level.

### Expression pattern and localization analysis of *FaTPK1*

To explore whether *FaTPK1* is involved in strawberry fruit ripening, the expression pattern of *FaTPK1* over the seven developmental fruit stages was determined using transcriptome data and SqRT–PCR. During fruit enlargement and de‐greening, *FaTPK1* transcripts decreased slowly from the SG to DG stages, and reached the lowest level in the Wt stage. Then, with the red fruit colour development, *FaTPK1* transcripts increased rapidly and reached their highest level in the FR stage (Figure 2d).

**Figure 3:**
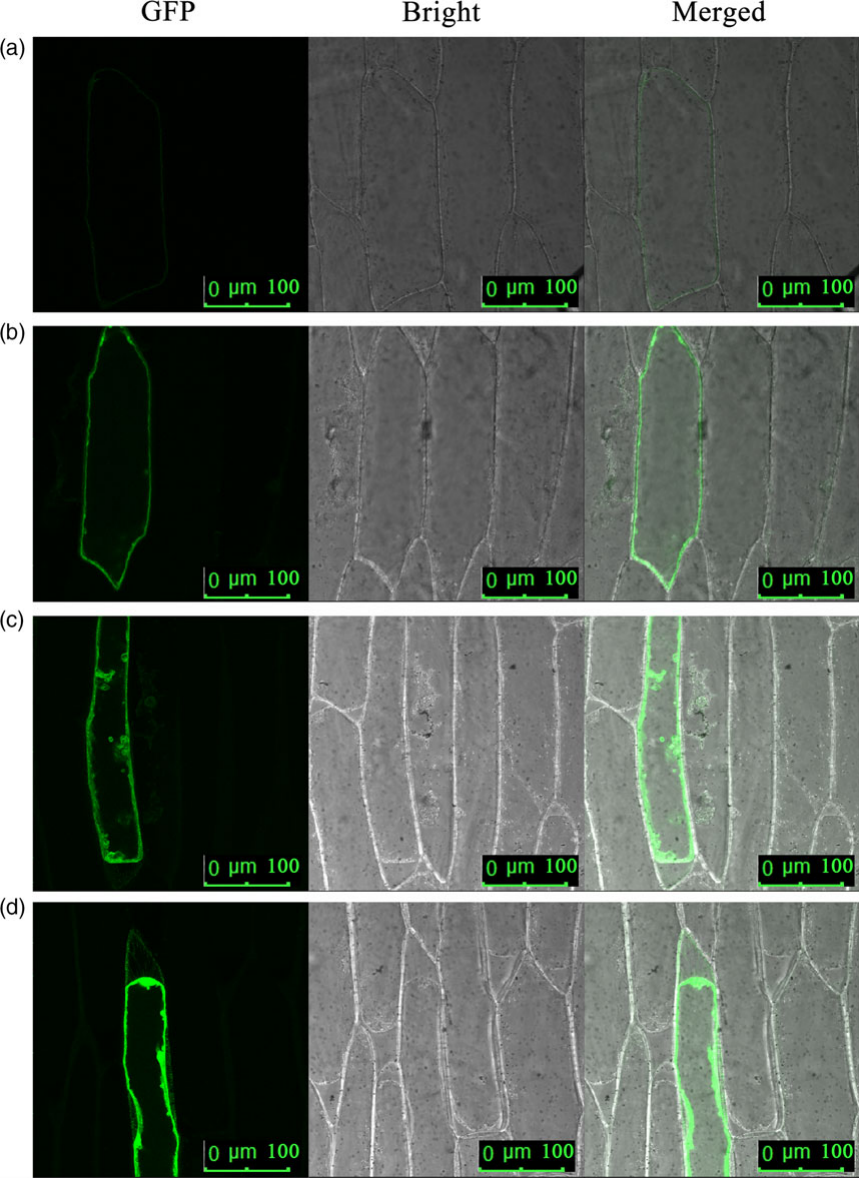
Subcellular localization analysis of *FaTPK1* in onion epidermis cells via Particle Bombardment. Onion epidermis cells were stably transformed with plasmid constructs cauliflower mosaic virus (CaMV) 35S: FaTPK1::EGFP.

An enhanced green fluorescence protein (EGFP)–FaTPK1 fusion protein in onion epidermis cells revealed that the transiently expressed fusion protein was targeted to the tonoplasts (Figure 3), indicating that *FaTPK1* was localized on the vacuole membrane.

### Functional analysis of *FaTPK1* in fruit

**Figure 4:**
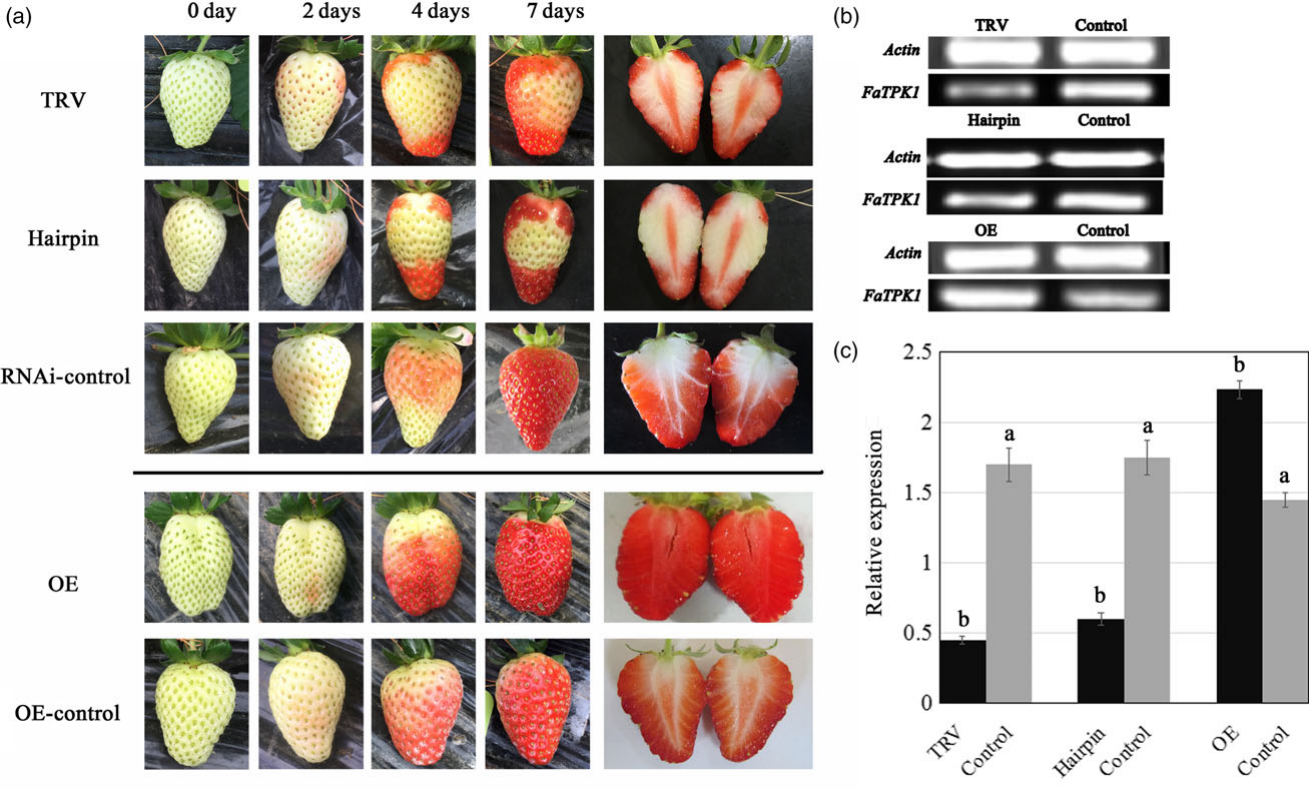
Phenotypes of *FaTPK1‐* RNA interference (RNAi) and *FaTPK1‐*overexpression (OE) strawberry fruits. (a) DG stage fruit attached to plants was infiltrated by GV3101. The TRV vector carrying a fragment of *FaTPK1* RNAi, an ihpRNAi construct based on a 650‐bp fragment of the *FaTPK1* coding region, was cloned into pFGC5941 vector. The *FaTPK1‐* OE construct was made via cloning *FaTPK1* into pCAMBIA1304. Expression of the target gene is controlled through a CaMV 35S promoter. Empty vector injection was used as the control. (b) SqRT–PCR analysis of *FaTPK1* expression in RNAi fruit and OE fruit. *Actin* was used as the internal control. (c) Quantitative reverse transcription–PCR analysis of *FaTPK1* expression in TRVs, ihpRNAi, OE and control fruits. Values are the means ± SD of four replicates. Columns with different letters (a, b) indicate statistically significant difference (*P* < 0.05) when the data are performed by variance analysis followed by Duncan's multiple range tests.

To investigate the role of *FaTPK1* in strawberry fruit development, we generated VIGS fruit (Jia *et al*., 2011) ihpRNA fruit (Hoffmann *et al*., 2006), and overexpression (OE) fruits. *Agrobacterium tumefaciens* strain GV3101 cultures containing pTRV1 and pTRV2‐FaTPK1 in a 1:1 ratio was infiltrated into DG fruits, and the control fruits were infiltrated with TRV empty vector alone. Seven days after infiltration, the surface of the control fruits turned fully red, while, in contrast, the inoculated sector on the surface of the RNA interference (RNAi) fruits remained white (Figure 4a, TRV), which was accompanied by a decrease in *FaTPK1* transcripts (Figure 4b,c, TRV).

An ihpRNAi construct of *FaTPK1* using a 650‐bp *FaTPK1* gene was cloned into pFGC5941 vector. The RNAi transient expression of *FaTPK1* was made by injecting the *Agrobacterium tumefaciens* strain GV3101 carrying *FaTPK1‐*RNAi into the DG fruits. The injection of empty pFGC5941 vector was adopted as control. The down‐regulation of *FaTPK1* expression (Figure 4b,c, hairpin) inhibited the *FaTPK1*‐RNAi fruit's red colour development (Figure 4a, hairpin).

A *FaTPK1‐*OE construct was created by cloning the coding sequence of *FaTPK1* into pCAMBIA1304 vector at the KpnI and EcoRI restriction sites. Expression of the target gene is controlled through a CaMV 35S promoter. After injection of the *FaTPK1‐*OE construct and the control vector of empty pCAMBIA1304 into the DG fruits, respectively. The mRNA expression levels of *FaTPK1* and the phenotypes of strawberry are shown in Figure 4; the up‐regulation of *FaTPK1* expression (Figure 4b,c, OE) accelerated the fruit's red colour development (Figure 4a, OE) compared with the control (Figure 4a, control). Thus, *FaTPK1* plays a role in fruit ripening.

### Altered *FaTPK1* expression affects the ripening‐related physiological processes

**Figure 5:**
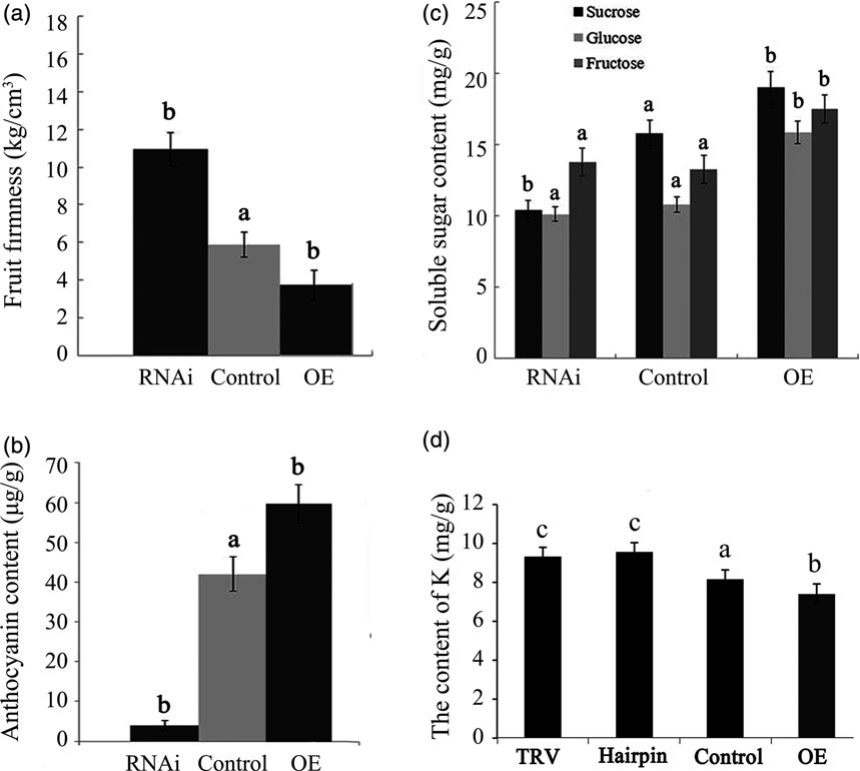
Changes in several physiological parameters and the ripening‐related gene transcripts in *FaTPK1*‐RNA interference (RNAi) and *FaTPK1‐*overexpression (OE) fruits. Changes in (a) firmness; (b) anthocyanin contents; (c) soluble sugar contents; and (d) Potassium contents in RNAi fruit, control fruit and OE fruit, respectively. Error bars represent the SE (*n* = 3). Columns with different letters (a, b) indicate statistically significant difference (*P* < 0.05) when the data are performed by variance analysis followed by Duncan's multiple range tests.

**Figure 6:**
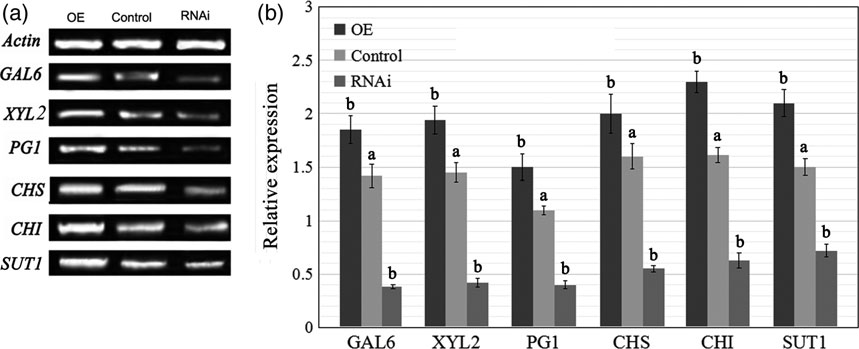
The effects of FaTPK1 RNAi and OE on transcripts of ripening‐related genes. (a) Changes in transcripts of ripening‐related genes *GAL6*, *XYL2*, *PG1*, *CHS*, *CHI* and *SUT1* were made by SqRT‐PCR. *Actin* was used as the internal control. Error bars represent the SE (*n* = 3). (b) Gene expression was examined by qPCR after transformation for 7 days. *Actin* was used as the internal control. Values are the means ± SD of three replicates. Columns with different letters (a, b) indicate statistically significant difference (*P* < 0.05) when the data are performed by variance analysis followed by Duncan's multiple range tests.

To further understand the role of *FaTPK1* in strawberry fruit ripening, we analysed the physiological conditions of fruit firmness, soluble sugar (glucose, fructose, and sucrose) contents and anthocyanin contents in transgenic fruits in which *FaTPK1* was down‐regulated and up‐regulated compared with the control fruits. Transcripts of genes regulated to these physiological processes: polygalacturonase (*PG1*), beta‐galactosidase (*GAL6*) and D‐xylulose reductase (*XYL2*) for fruit firmness; sucrose transporter (*SUT1*) for sugars; and chalcone synthase (*CHS*) and chalcone flavanone isomerase (*CHI*) for anthocyanins (Jia *et al*., 2011, 2013; Tian *et al*., 2012) were also assessed. The fruit firmness increased in RNAi fruits but declined in OE fruits (Figure 5a), whereas the anthocyanin (Figure 5b) and sugar (Figure 5c) contents were down‐regulated in RNAi fruit and up‐regulated in OE fruit compared with the control fruits. The K^+^ contents were determined using the inductively coupled plasma mass spectrometry method. The tonoplast *FaTPK1* is an outward potassium channel and controls K^+^ release from vacuoles; during strawberry fruit ripening, K^+^ was maintained at a high level. In the RNAi, control and OE fruits, there was a declining trend in the K^+^ content (Figure 5d) with sugar accumulation (Figure 5c). The SqRT–PCR (Figure 6a) and qPCR (Figure 6b) analysis showed that the mRNA expression levels of all of the tested genes were down‐regulated in RNAi fruits and up‐regulated in OE fruits (Figure 6). The changes in transcripts of firmness‐ (*PG1, XYL2* and *GAL6*), sugar‐ (*SUT1*) and pigment‐related (*CHS* and *CHI*) genes in these transgenic fruits were accordant to a role of *FaTPK1* in ripening.

### Altered *FaTPK1* expression affects fruit pathogen resistance

**Figure 7:**
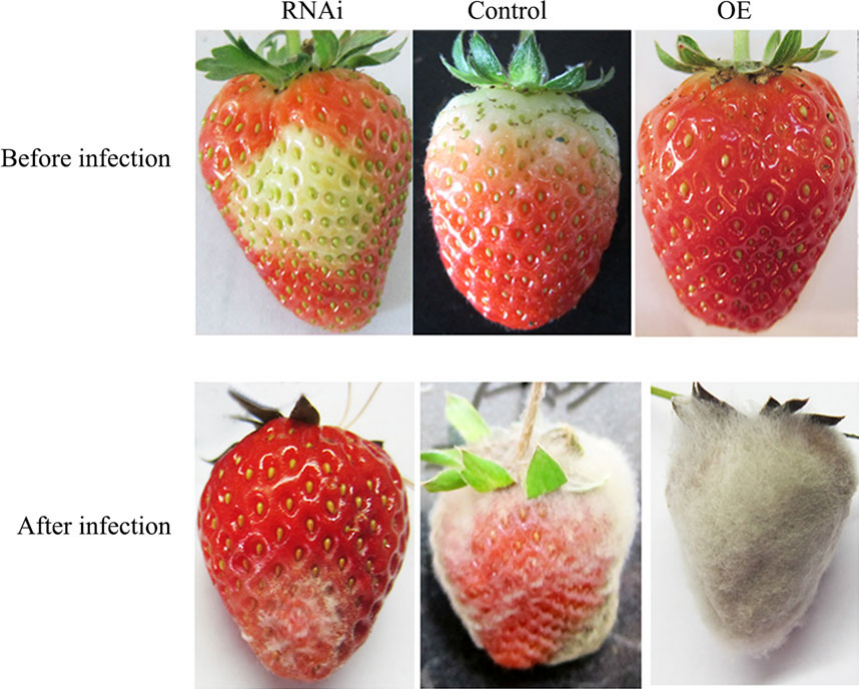
Alteration of *FaTPK1* expression affects the fruit's pathogen resistance. The fungal spore concentration was 10^6^ CFU/mL. Five days after infection, the FaTPK1‐RNAi fruit were more resistant to *Botrytis cinerea* than control fruits, but the FaTPK1‐OE fruits were more sensitive to the pathogen than control fruits.

Ripened fruit is susceptible to pathogens, which limits the fruit storage periods and preservation. To explore the role of *FaTPK1* in pathogen resistance, *B. cinerea* was used to infect strawberry fruits. Five days after inoculation, the degree of infection was divided into four grades: level one (no infection), level two (20% infection), level three (60% infection) and level four (100% infection). As shown in Figure 7, fruits infected with 10^6 ^CFU/mL spores exhibited *B. cinerea* lesions at 5 days after inoculation, while the *FaTPK1*‐RNAi fruits appeared only lightly infected. By contrast, the control fruits had moderate infection levels and the *FaTPK1*‐OE fruits were severely infected at 5 days after inoculation. The disease indices of the *FaTPK1*‐RNAi, control and *FaTPK1*‐OE fruits were 51.67, 76.67 and 96.67, respectively (Table 1). Thus, *FaTPK1*‐RNAi fruits were significantly resistant to *B. cinerea* compared to the control, and, in contrast, *FaTPK1*‐OE fruits were sensitive to the pathogens, indicating that *FaTPK1* is involved in pathogen resistance.

**Table 1:** Statistics on the number of fungi *Botrytis cinerea*‐infected fruits

Infection degree of *Botrytis cinerea*	*FaTPK1‐RNAi* fruits (number)	Morbidity	Control fruits (number)	Morbidity	*FaTPK1‐OE* fruits (number)	Morbidity
Level one	0	0	0	0	0	0
Level two	14	93.30%	1	0.067%	0	0
Level three	1	0.067%	12	80%	2	0.133%
Level four	0	0	2	0.133%	13	86.70%
Disease index	51.6		76.67		96.67	

Note: The data were the average value of three replicates.

### Analysis of the FaTPK1 protein using ITC

**Figure 8:**
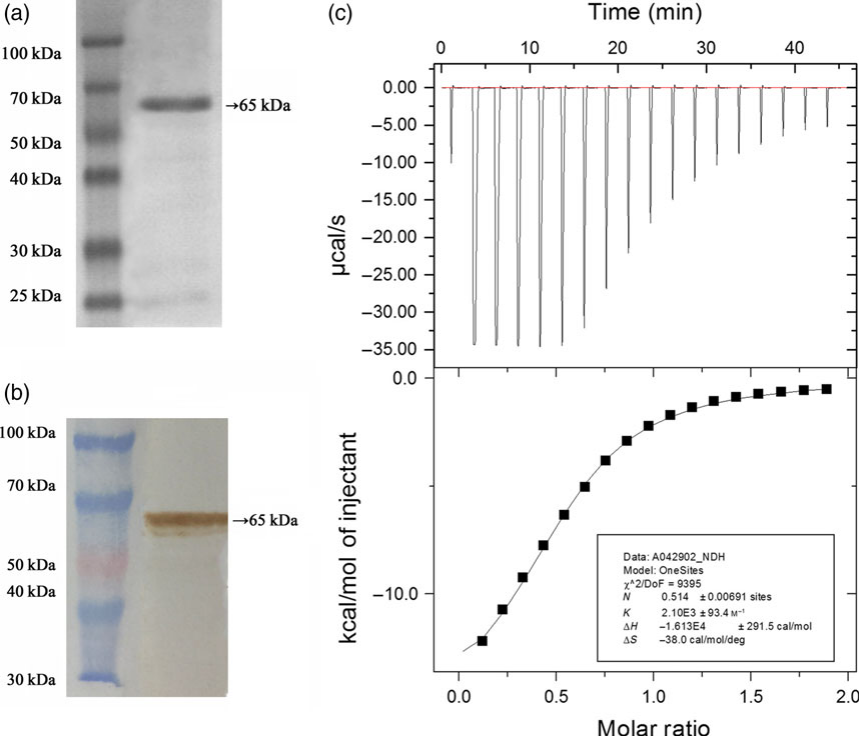
Purification of the FaTPK1 fusion protein. (a) The 65‐kDa fusion protein of *FaTPK1* was collected using a GST tag. The molecular weight marker is 25–100 kDa. (b) Identification of the FaTPK1 fusion protein using a one‐step western kit with horseradish peroxidase (Mouse). The protein expression increased after induction with 1 mm IPTG. (c) The binding of K^+^ with the purified FaTPK1 protein ITC.

To further explore the binding capacity of the FaTPK1 protein with K^+^, the coding sequence of *FaTPK1* was amplified and expressed in *Escherichia coli* cells, and 1.2 mg/mL of a ~65‐kD recombinant fusion protein was purified using Beaver Beads™ GHS (Glutathione S‐transferase) tag (Figure 8a). The protein was identified using a one‐step western kit with horseradish peroxidase (Figure 8b). Then, an ITC analysis was performed using 20 μm of FaTPK1 recombinant protein and 100 mm of KCl in an ITC200 calorimeter, in which the protein samples were titrated with a 2‐μL injection of KCl per 150 s. The KCl–buffer–FaTPK1 recombinant protein titration system showed that the binding of KCl to FaTPK1 followed a saturation kinetics curve with a binding constant of 2.1 × 10^‐3^ m ^−1^ and a dissociation constant of 476 μm (Figure 8c). The control results gained from the (Tris–HCl) buffered protein did not follow a saturation curve (data not shown). Thus, the FaTPK1 protein could bind K^+^.

### Down‐regulation and overexpression of the *FaTPK1* gene alter the ABA content

**Figure 9:**
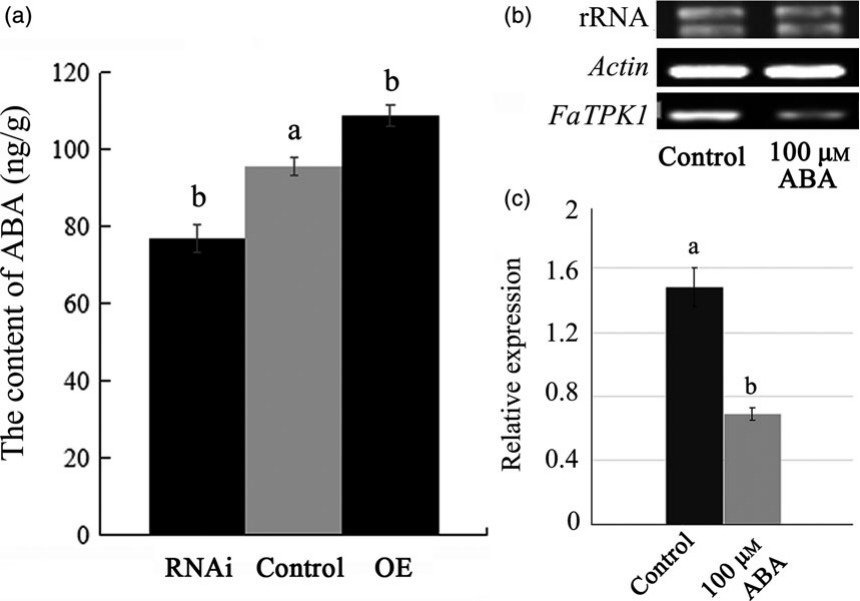
Relationship of ABA with transcripts of *FaTPK1*. (a) Changes of *FaTPK1* transcripts affect ABA level in RNA interference (RNAi) and overexpression (OE) fruits. In RNAi fruits, the ABA contents decreased, while in OE fruits, the ABA contents increased compared with control fruits. (b) ABA can inhibit the transcription of *FaTPK1* in vivo. Two‐week‐old strawberry fruits were treated by 100 μm ABA in vivo. After 6 days, the phenotypes were investigated. Error bars represent the SE (*n* = 3). (c) qPCR analysis of *FaTPK1* expression in the control and ABA‐treated fruits. Values are the means ± SD of three samples. Columns with different letters (a, b) indicate statistically significant difference (*P* < 0.05) when the data are performed by variance analysis followed by Duncan's multiple range tests.

To investigate a relationship of *FaTPK1* with ABA during ripening, the ABA content in the *FaTPK1*‐RNAi fruits decreased compared with the control fruits, while it increased in the OE fruits. The average ABA contents were 76.816 ± 3.546 ng/g, 108.877 ± 2.752 ng/g and 95.689 ± 2.378 ng/g in RNAi, OE and control fruits, respectively. Thus, the ABA content was significantly down‐regulated in infiltrated RNAi fruits but significantly up‐regulated in the OE fruits compared to the control fruits (Figure 9a). ABA can inhibit *FaTPK1* transcription in vitro, and when exogenous ABA was sprayed on intact strawberry fruit, the *FaTPK1* expression decreased (Figure 9b,c). Thus, ABA could inhibit *FaTPK1* transcription.

## Discussion

The developmental processes of fleshy fruits have three distinct, overlapping phases: cell division (early stage), cell expansion (middle stage) and ripening (late stage). Most of the cell's volume is occupied by a large central vacuole, and its osmotic‐driven enlargement determines the fruit's size and development (Ho, 1996). An H^+^‐coupled accumulation of soluble sugars localizes predominantly in the vacuole throughout tomato fruit growth, and the osmotic strength of the vacuole is highly controlled by sucrose import during fruit development (Beauvoit *et al*., 2014).

Tonoplast *TPK1* mediates K^+^‐selective currents between the cytoplasm and vacuole. *TPK1* activity is regulated by cytoplasmic Ca^2+^ and the cytoplasmic pH. *TPK1* is also involved in the translocation of intracellular K^+^ and the redistribution of K^+^ among the different plant tissues (Gobert *et al*., 2007). The variation in the K^+^ distribution of different genotypes may result from differences in *TPK1* expression. Gobert *et al*. (2007) also found that K^+^ release is delayed in the presence of ABA when the *TPK1* gene is knocked out, which can inhibit plant growth because of a slower stomatal closure rate. This suggests that *TPK1* could be a possible pathway for vacuolar K^+^ release during stomatal closure in the presence of ABA.

The rapid accumulation of apoplast‐unloaded sucrose and subsequent sucrose synthase‐mediated sucrolysis resulted in an increase in the contents of glucose, fructose and especially sucrose, which promoted strawberry fruit ripening (Jia *et al*., 2011; Li *et al*., 2012a,b; Tian *et al*., 2012; Zhao *et al*., 2017). Here, we showed that the soluble sugar contents rapidly and continually increased during fruit ripening (Figure 1). The contents of the soluble sugars were down‐regulated in the *FaTPK1*‐RNAi fruits but up‐regulated in the *FaTPK1*‐OE fruits (Figure 5), suggesting that a link may be present between *FaTPK1* and soluble sugar accumulation, especially sucrose accumulation.

The vacuole‐mediated K^+^ uptake and efflux are essential and inevitable for plant growth and development, as well as stress responses, because they control the cell water potential and turgor during osmotic regulation (Latz *et al*., 2007; Nieves‐Cordones *et al*., 2016; Osakabe *et al*., 2013; Sharma *et al*., 2013). In *Vicia faba* leaf guard cells, stomatal opening is correlated with more K^+^ uptake and less sucrose accumulation, and maximal apertures are coupled with a drastic decrease in the K^+^ content, while sucrose becomes the dominant solute (Talbott and Zeiger, 1996, 1998). In the present study, we showed that K^+^ is the most abundant element in strawberry fruit (Table S1), and its contents, on the whole, showed a declining and oscillating trend during fruit development (Figure 1). It was up‐regulated in *FaTPK1*‐RNAi fruits but down‐regulated in *FaTPK1*‐OE fruits (Figure 5), demonstrating that fruit ripening is coupled with a rapidly accumulating sucrose content and a decrease in the K^+^ contents. Thus, *FaTPK1* can regulate the K^+^ contents, which modulates sucrose metabolism and affects fruit firmness, anthocyanin content and soluble sugar accumulation, as well as the biotic stress responses and the transcriptional levels of the ripening‐regulated genes *GAL6*,* XYL2*,* PG1*,* CHS*,* CHI* and *SUT1* (Figure 6). Previous study demonstrated that K^+^ is released rapidly from the vacuole (Macrobbie, 2006) after the application of ABA, which may play a role in the ripening of nonclimatic fruit (Jia *et al*., 2011). Thus, ABA can affect the activity of *FaTPK1*. In our study, the ABA contents were down‐regulated in *FaTPK1*‐RNAi fruits and up‐regulated in *FaTPK1*‐OE fruits compared to control (Figure 8a). This suggested that silencing *FaTPK1* could inhibit fruit ripening but overexpressing it could promote fruit ripening. Thus, our results indicate that *FaTPK1*‐mediated K^+^ oscillation plays a role in soluble sugar accumulation and, as a result, regulates fruit ripening and quality.

It is known that soluble sugar main stored in vacuoles of ripened fleshy fruit. The strawberry K^+^ channel FaKAT1 plays a role in sugar absorption through cell membranes (Song *et al*., 2017). In present study, we provided a set of physiological, chemical and molecular evidence to demonstrate that FaTPK1 plays a role in sugar absorption through tonoplast membrane. Given that ripened strawberry fruit mainly accumulates sucrose (Jia *et al*., 2011) and sucrose is a signal molecule in strawberry fruit ripening (Jia *et al*., 2013). Thus, the mechanism of coordinated regulation of FaKAT1, FaTPK1 and sucrose in sugar accumulation is interesting work in the future. *FaTPK1*, a K^+^ channel on tonoplasts, has the function of controlling K^+^ effluxes from vacuoles. Thus, the efflux of K^+^ may promote the accumulation of sucrose in strawberry ripening.

## Experimental procedures

### Plant materials

In this study, strawberry ‘Sweet charlie’ (*Fragaria × ananassa*) fruits were used. Strawberry plants were cultivated in greenhouse at 23–28 °C, with 60%–70% relative humidity. Fruits were classified into seven developmental stages: SG, BG, DG, Wt, IR, PR and FR, which were collected at 7, 14, 18, 21, 23, 25 and 28 days after anthesis, respectively. Twenty fruits of uniform size were sampled at every stage (one replication). Fresh fruit (nonseed fruit tissue) was used to measure the physiological indices.

### RNA‐seq and data analysis

Four stages (BG, Wt, IR and PR) of fruits (*n* = 3) were sampled for RNA isolation and cDNA synthesis. RNA was extracted from each receptacle using E.Z.N.A. Total RNA Kit (Omega Bio‐tek, Norcross, GA), then the RNA was used for cDNA library synthesis after RNase‐free DNase digestion based on a RNA library prep kit (New England BioLabs, Ipswich, MA). RNA‐seq was performed on the Illumina HiSeq2000 platform (Illumina, San Diego, CA) by Beijing Ori‐Gene Science and Technology Corp., LTD, and analysis of the RNA‐seq data was carried out according the report (Benjamini and Yekutieli, 2001; Langmead and Salzberg, 2012; Mortazavi *et al*., 2008; Wang *et al*., 2010; Zhao *et al*., 2017).

### Cloning of *FaTPK1* gene

To clone the *FaTPK1* gene, the specific primers (forward, 5′‐ATGGATAGGAATGGGATGCG‐3′; reverse, 5′‐TTATGATTGAGCAAGTGTTAGAT‐3′) were designed. The *FaTPK1* cloning was performed on according to the description as the following PCR conditions: 98 °C for 30 s, 98 °C for 10 s (35 cycles), 55 °C for 30 s, 72 °C for 40 s and with a final extension of 72 °C for 2 min in 50 μL Q5 PCR mixture (0.5 μL Q5 high‐fidelity DNA Polymerases, 10 μL 5 × Q5 buffer, 1 μL 10 mm dNTPs, 2.5 μL forward specific primer, 2.5 μL reverse specific primer, 2 μL cDNA template, 31.5 μL ddH_2_O). The PCR products were ligated into T1‐simple cloning vector and subsequently transformed into *Escherichia coli* competent cell (TransGen Biotech, Beijing, China). Positive colonies were selected and sequenced by Huada China (Beijing, China).

### Construction of recombinant plasmids

Using VIGS (Liu *et al*., 2002), *FaTPK1* was silenced in strawberry fruit. A 426‐bp cDNA fragment of *FaTPK1* was amplified using primers (sense, 5′‐GGAATTCATGGATAGGAATGGGATGCG‐3′; antisense, 5′‐GGGGTACCAATAGTCTGCTGCTTGGCTCA‐3′) by PCR, and the DNA fragments were cloned into *Kpn*I–*Eco*RI‐cut virus vector pTRV2. *Agrobacterium*‐mediated TRV infection was made as described by Fu *et al*. (2005).

To generate ihpRNAi of *FaTPK1*, a 650‐bp fragment of the *FaTPK1* cDNA was PCR amplified using the primers (sense, 5′‐CGGGATCCATGGATAGGAATGGGATGCG‐3′; antisense, 5′‐CATGCCATGG TAAAATGCATCCACT AGGTC‐3′). The amplified fragment was cloned into pFGC5941 vector (Mubin *et al*., 2011) at the *Nco*I and *Bam*HI restriction sites, and the recombinant plasmid was named pFGC650. A 350‐bp fragment, which complementary to the 3′ end of the 650‐bp fragment, was amplified by PCR using specific primers (sense, 5′‐CGGGATCCGCTGTTTATTT CTGTATTGT‐3′ and antisense, 5′‐GCTCTAGATAAAATGCATCCACTAGGTCCAAT‐3′) from strawberry fruit cDNA. The 350‐bp fragment was cloned into pFGC650 at *Bam*HI and *Xba*I restriction sites. By this means, a hairpin structure was produced in the recombinant plasmid.

To generate the *FaTPK1* OE construct, the full‐length of *FaTPK1* was obtained by PCR from cDNA using specific primers (sense, 5′‐GG GGTACCATGGATAGGAATGGGATGCG‐3′; antisense, 5′‐G GAATTC TTATGATTGAGAAGTGTTAGAT‐3′). The cDNA was cloned into the pCAMBIA1304 vector at the *Kpn*I and *Eco*RI restriction sites.

### Transfection of strawberry by agroinfiltration

*Agrobacterium tumefaciens* strain GV3101, containing pTRV2‐*FaTPK1*, pFGC5941‐*FaTPK1* and pCAMBIA1304‐*FaTPK1*, was cultured in Luria–Bertani liquid medium at 28 °C containing 10 mm 2‐(N‐morpholino) ethanesulfonic acid (MES) and 20 μm acetosyringone with appropriate antibiotics. Vectors pTRV2, pFGC5941 and pCAMBIA1304 were the controls. The *Agrobacterium* cells were cultured to an optical density at 600 nm (OD_600_) of 0.8 and *Agrobacterium* infection as described by Chai *et al*. (2011) and Jia *et al*. (2013). Strawberry fruits were treated by local and whole fruit injection with 200 μL and 1 mL of the *Agrobacterium* suspension, respectively.

### SqRT–PCR and SYBR real‐time PCR

In each treatment group, strawberry fruits (*n* = 6) growing at the same rate were acquired using the random sampling method. For analysis of *FaTPK1* transcripts by SqRT–PCR in RNAi and OE fruits, six fruits were randomly selected for mixed grinding. First‐strand cDNA was used as the template for 28 cycles of PCR amplification of *FaTPK1* using 20 μL PCR mixture. The reactions contained 0.2 μL LATaq (5U/μL), 2 μL 10 × LA PCR buffer II (Mg^2+^ plus), 3.2 μL dNTP Mixture (2.5 mm), 1 μL forward specific primer (10 μm; Sangon, Shanghai, China), 1 μL reverse specific primer (10 μm; Sangon), 1 μL cDNA template and 11.6 μL ddH_2_O. The experiment above was repeated three times. A 418‐bp *FaTPK1* was amplified by primers (sense, 5′‐TGCTTCCAGGGGTCATAG‐3′; and antisense, 5′‐AGTCTGCTGCTTGG CTCA‐3′). These conditions were selected for the comparison of the relative accumulation of *FaTPK1* and *Actin*. After silence and overexpression of *FaTPK1* in strawberry, the ripening‐regulated genes *GAL6*,* XYL2*,* PG1*,* CHS*,* TPK1*,* CHI* and *SUT1* were investigated by SqRT–PCR, primers as shown in Table S2. The primers used for SYBR real‐time PCR are showed in Table S3 and made according to the description by Chai *et al*. (2011). The experiment was performed with three replications.

### Expression and purification of the FaTPK1 recombinant protein

The expression and purification of the recombinant FaTPK1 protein was performed using a prokaryotic expression system in *E. coli*. The coding sequence of *FaTPK1* was amplified by PCR from a synthesized cDNA using primers forward, 5′‐CCGGAATTCATGGATAGGAATGGGATGCG‐3′ (*Eco*RI site); and reverse, 5′‐ATAAGAAT GCGGCCGCATTATGATTGAGCAAGTGTTA‐3′ (*NOT*I site) and cloned into the expression vector PGEX‐4T1 in frame with the N‐terminal GST fusion tag, which was transformed into *E. coli* to enable the selection of transformants on LB plates containing 100 μg/mL ampicillin. Ten transformants were selected to confirm the correct fusion frame by sequencing. The purified recombinant plasmids were transformed into *E. coli* BL21 to select the ampicillin‐resistant *E. coli* transformants. The FaTPK1‐GST fusion protein was expressed at 16 °C in LB broth with 1 mm IPTG for 12 h. The purification of the FaTPK1‐GST fusion protein was carried using BeaverBeads™ GHS (Beaver, China) according to manufacturer's protocols. The eluted fusion protein was stored at −80 °C until use.

### Subcellular localization of FaTPK1 in onion inner epidermal cells

The *FaTPK1* was cloned into the pEZS‐NL vector (Li *et al*., 2012a,b) using primers forward, 5′‐G GAATTC ATGGATAGGA ATGGGATGCG‐3′ (*Eco*RI), reverse, 5′‐GG GGTACC TTATGATTGAGCAAGTGTTA‐3′ (*Kpn*I) to obtain an EGFP‐fused protein. The plasmids were transformed into onion inner epidermal cells by particle bombardment using biolistic delivery of gold particles (Bio‐Rad, PDS1000/He Biolistic Gene Gun, Hercules, CA) to get the subcellular localization information of the expressed proteins. The effective bombardment parameters were 1100 Psi of helium pressure, 84.66 kPa of vacuum pressure and 6 cm of target‐shelf distance, and the amount of gold powder for is 0.7 mg. The onion epidermis was incubated for 4 h on MS plates (sucrose 30 g/L, sorbitol 0.15 m, mannitol 0.15 m, agar 8 g/L, pH 5.8). After bombardment, the onion epidermis cells were incubated for 24 h on MS plates (sucrose 30 g/L, agar 8 g/L, pH 5.8). After incubation at 25 °C for 24 h, the fluorescence of EGFP was observed using a confocal laser scanning microscope (Zeiss LSM 510 META, Germany) with excitation at 488 nm. Confocal observations were made using a Plan‐Apochromat X40 dry objective. The experiment was performed with three replications.

### ITC assay

The eluted fusion protein concentration was used an Amicon Ultra‐4 centrifugal 30‐kDa filter (Millipore, Darmstadt, Germany). The FaTPK1 was adjusted to 20 μm in ITC buffer (50 mm Tris‐HCl, pH 8.0). The protein samples were titrated using a 2‐μL injection with 100 mm of KCl per 150 s in Microcal iTC200 (Malvern, Worcestershire, UK) at 25 °C. The titration of Tris–HCl buffer into the FaTPK1 protein was used as the control. The experiment was repeated three times.

### Determination of mineral element contents by inductively coupled plasma mass spectrometry method

To determine the mineral element contents of strawberry in the seven developmental stages, six RNAi, OE and control fruits (control fruits for RNAi and OE were mixed) were randomly selected for mixed grinding, respectively. For RNAi fruits, we selected fruits that had delayed maturity by 50% and selected OE fruits that promoted maturity by 200%. Samples (0.5 g, *n* = 3) were digested in 5 mL of HNO_3_ and 4 mL of H_2_O_2_ at 16 °C using a microwave digestion instrument (MARS‐240/50). After cooling samples to room temperature, they were dried by electrothermal heating and their volumes increased to 50 mL. The solvents were used as controls. The mineral element contents of strawberry fruits were determined by inductively coupled plasma mass spectrometry (ICAP6300 Radial, Thermo Fisher, Waltham, MA). Three replications were performed.

### Determination of the soluble sugar and ABA content

The soluble sugar content was determined using reverse phase HPLC (Agilent Technologies 1200 Series, RID1 A detector). The supernatant was fractionated using a Sugar‐Pak™1 column (6.5 × 300 mm, Waters) with 100% MilliQ water for 25 min at a flow rate of 0.4 mL per min. The column temperature was 80 °C, and the injection volume was 20 μL. Standard samples used were D‐(+) glucose, D‐(–) fructose and sucrose (Sigma‐Aldrich, St. Louis, MO).

Half of the RNAi, control and OE strawberry fruits with fresh infections were used for sampling. According to the method described by Song *et al*. (2017), ABA was extracted and stored at –20 °C. An enzyme‐linked immune sorbent assay was used for ABA content determination as described by Zhang *et al*. (2009). The experiment was repeated three times.

### Determination of the anthocyanin content

Ripening strawberry fruits (*n* = 10) of consistent maturity were selected and treated by liquid nitrogen grinding. Then, 2.5 mL of methanol (1% HCl) was added to 0.5 g of powder, extracted overnight in the darkness at 4 °C, after centrifugation at 10,000 g for 10 min, carefully collect the supernatant into a 10‐ml triangular flask. The residues were mixed with 2.5 mL of methanol (1% HCl) and then centrifuged as described above. The volume was then increased to 10 mL with distilled water, and 1 mL extract was filtered with a 0.22 micron membrane; the filtrate was used for the determination of the anthocyanin content.

The anthocyanin content was measured by reverse phase HPLC using a ZORBAX Eclipse XDB‐C18 column (4.6 × 150 mm, 5 μm, Agilent) with a linear gradient from solution A (acetonitrile), 0 to 20% for 13 min, 20% to 40% for 20 min and 0% for 25 min, to solution B (10% formic acid) at a flow rate of 1 mL per min. The detection wavelength was 520 nm, the column temperature was 25 °C, and the injection volume was 20 μL. The standard sample used was pelargonidin‐3‐O‐glucoside. The entire process was repeated three times.

### Effect of ABA on strawberry fruit ripening in vivo

Wt stage fruits were selected and immersed into 100 μm of ABA (a treatment includes 20 fruits per replication), and ddH_2_O was used as the control. The developing fruits still attached to plants were treated four times on alternating days beginning at 14 days after anthesis. Whole strawberry fruits treated with 100 μm ABA or ddH_2_O were used for sampling. Seven days after the first treatment, the expression level of *FaTPK1* was investigated by sqRT–PCR and qPCR.

### Fruits infection with *B. cinerea*

Culture of fungi (*B. cinerea*) on PDA plating medium with the temperature at 25 °C and the relative humidity reached to 95%. The spores were eluted from the culture medium after 8 days. After removing mycelia, spores were counted and added to the inoculation solution at the concentration of 10^6 ^CFU/mL. The *FaTPK1*‐RNAi strawberry fruits (*n* = 15) and *FaTPK1*‐OE fruits (*n* = 15) were incubated in an inoculation solution containing 10^6 ^CFU/mL spores for 5 min. Then, the strawberry fruits were put into incubator at 25 °C, and the incubators were covered with plastic film to guarantee a relative humidity of 95%–100%. The control fruits (*n* = 15) were treated with sterile water. After inoculation for 5 days, we count the number of spreading lesions on each fruit to evaluate fruit infection caused by *B. Cinerea* (Li *et al*., 2013).

## Supporting information


**Figure S1** Nucleotide blast of *FaTPK1* gene and *FvTPK1* gene.
**Table S1** Element contents in ripening fruits (FR) and 7‐stage fruits (average)
**Table S2** The primers used for SqRT‐PCR
**Table S3** The primers used for qPCR

## References

[pbi12824-bib-0001] Ahmad, I. , Devonshire, J. , Mohamed, R. , Schultze, M. and Maathuis, F.J. (2016) Overexpression of the potassium channel TPKb in small vacuoles confers osmotic and drought tolerance to rice. New Phytol. 209, 1040–1048. 26474307 10.1111/nph.13708

[pbi12824-bib-0002] Allen, G.J. and Sanders, D. (1995) Calcineurin, a type 2B protein phosphatase, modulates the Ca2+ ‐permeable slow vacuolar ion channel of stomatal guard cells. Plant Cell 7, 1473–1483. 12242407 10.1105/tpc.7.9.1473 PMC160973

[pbi12824-bib-0003] Beauvoit, B.P. , Colombié, S. , Monier, A. , Andrieu, M.H. , Biais, B. , Bénard, C. , Chéniclet, C. *et al*. (2014) Model‐assisted analysis of sugar metabolism throughout tomato fruit development reveals enzyme and carrier properties in relation to vacuole expansion. Plant Cell 26, 3224–3242. 25139005 10.1105/tpc.114.127761 PMC4371827

[pbi12824-bib-0004] Benjamini, Y. and Yekutieli, D. (2001) The control of the false discovery rate in multiple testing under dependency. Ann. Stat. 29, 1165–1188.

[pbi12824-bib-0005] Chai, Y.M. , Jia, H.F. , Li, C.L. , Dong, Q.H. and Shen, Y.Y. (2011) FaPYR1 is involved in strawberry fruit ripening. Plant Growth Regul. 62, 5079–5089. 10.1093/jxb/err207 21778181

[pbi12824-bib-0006] Conde, C. , Silva, P. , Fontes, N. , Dias, A.C.P. , Rui, M.T. , Sousa, M.J. , Agasse, A. *et al*. (2006) Biochemical changes throughout grape berry development and fruit and wine quality. Food 1, 1–22. *Structure, 1*.

[pbi12824-bib-0007] Enyedi, P. and Czirjak, G. (2010) Molecular background of leak K+ currents: two‐pore domain potassium channels. Physiol. Rev. 90, 559–605. 20393194 10.1152/physrev.00029.2009

[pbi12824-bib-0008] Fu, D.Q. , Zhu, B.Z. , Zhu, H.L. , Jiang, W.B. and Luo, Y.B. (2005) Virus‐induced gene silencing in tomato fruit. Plant J. 43, 299–308. 15998315 10.1111/j.1365-313X.2005.02441.x

[pbi12824-bib-0009] Given, N.K. , Venis, M.A. and Gierson, D. (1988) Hormonal regulation of ripening in the strawberry, a non‐climacteric fruit. Planta 174, 402–406. 24221523 10.1007/BF00959527

[pbi12824-bib-0010] Gobert, A. , Isayenkov, S. , Voelker, C. , Czempinski, K. and Maathuis, F.J. (2007) The two‐pore channel TPK1 gene encodes the vacuolar K+ conductance and plays a role in K+ homeostasis. PNAS 104, 10726–10731. 17563365 10.1073/pnas.0702595104 PMC1965580

[pbi12824-bib-0011] Hamamoto, S. , Marui, J. , Matsuoka, K. , Higashi, K. , Igarashi, K. , Nakagawa, T. , Kuroda, T. *et al*. (2008) Characterization of a tobacco TPK‐type K+ channel as a novel tonoplast K+ channel using yeast tonoplasts. J. Biol. Chem. 283, 1911–1920. 18029350 10.1074/jbc.M708213200

[pbi12824-bib-0012] Hedrich, R. (2012) Ion channels in plants. Physiol. Rev. 92, 1777–1811. 23073631 10.1152/physrev.00038.2011

[pbi12824-bib-0013] Ho, L.C. (1996) The mechanism of assimilate partitioning and carbohydrate compartmentation in fruit in relation to the quality and yield of tomato. J. Exp. Bot. 47, 1239–1243. 21245255 10.1093/jxb/47.Special_Issue.1239

[pbi12824-bib-0014] Hoffmann, T. , Kalinowski, G. and Schwab, W. (2006) RNAi‐induced silencing of gene expression in strawberry fruit (*Fragaria*× *ananassa*) by agroinfiltration: a rapid assay for gene function analysis. Plant J. 48, 818–826. 17092319 10.1111/j.1365-313X.2006.02913.x

[pbi12824-bib-0015] Isayenkov, S. , Isner, J.C. and Maathuis, F.J. (2011) Rice two‐pore K+ channels are expressed in different types of vacuoles. Plant Cell 23, 756–768. 21224427 10.1105/tpc.110.081463 PMC3077780

[pbi12824-bib-0016] Jia, H.F. , Chai, Y.M. , Li, C.L. , Lu, D. , Luo, J.J. , Qin, L. and Shen, Y.Y. (2011) Abscisic acid plays an important role in the regulation of strawberry fruit ripening. Plant Physiol. 157, 188–199. 21734113 10.1104/pp.111.177311 PMC3165869

[pbi12824-bib-0017] Jia, H.F. , Wang, Y.H. , Sun, M.Z. , Li, B.B. , Han, Y. , Zhao, Y.X. , Li, X.L. *et al*. (2013) Sucrose functions as a signal involved in the regulation of strawberry fruit development and ripening. New Phytol. 198, 453–465. 23425297 10.1111/nph.12176

[pbi12824-bib-0018] Langmead, B. and Salzberg, S.L. (2012) Fast gapped‐read alignment with bowtie 2. Nat. Methods 9, 357–359. 22388286 10.1038/nmeth.1923 PMC3322381

[pbi12824-bib-0019] Latz, A. , Becker, D. , Hekman, M. , Müller, T. , Beyhl, D. , Marten, I. , Bertl, A. *et al*. (2007) TPK1, a Ca2+ ‐regulated Arabidopsis vacuole two‐pore K+ channel is activated by 14‐3‐3 proteins. Plant J. 52, 449–459. 17764516 10.1111/j.1365-313X.2007.03255.x

[pbi12824-bib-0020] Lebaudy, A. , Véry, A.A. and Sentenac, H. (2007) K+ channel activity in plants: genes, regulations and functions. FEBS Lett. 581, 2357–2366. 17418142 10.1016/j.febslet.2007.03.058

[pbi12824-bib-0021] Li, C. , Jia, H. , Chai, Y. and Shen, Y. (2011) Abscisic acid perception and signaling transduction in strawberry: a model for non‐climacteric fruit ripening. Plant Signal Behav. 6, 1950–1953. 22095148 10.4161/psb.6.12.18024 PMC3337185

[pbi12824-bib-0022] Li, C.L. , Fang, K.F. , Lei, H. , Xing, Y. and Shen, Y.Y. (2012a) Phloem unloading follows an extensive apoplastic pathway in developing strawberry fruit. J. Hortic. Sci. 87, 470–477.

[pbi12824-bib-0023] Li, T.Y. , Wang, Y. , Zhang, X.Z. and Han, Z.H. (2012b) Isolation and characterization of ARRO‐1 genes from apple rootstocks in response to auxin treatment. Plant Mol. Biol. Rep. 30, 1408–1414.

[pbi12824-bib-0024] Li, Q. , Ji, K. , Sun, Y. , Luo, H. , Wang, H. and Leng, P. (2013) The role of FaBG3 in fruit ripening and *B. cinerea* fungal infection of strawberry. Plant J. 76, 24–35. 23802911 10.1111/tpj.12272

[pbi12824-bib-0025] Liu, Y.L. , Schiff, M. and Dinesh‐Kumar, S.P. (2002) Virus‐induced gene silencing in tomato. Plant J. 31, 777–786. 12220268 10.1046/j.1365-313x.2002.01394.x

[pbi12824-bib-0026] Maathuis, F.J. (2011) Vacuolar two‐pore K+ channels act as vacuolar osmosensors. New Phytol. 191, 84–91. 21371040 10.1111/j.1469-8137.2011.03664.x

[pbi12824-bib-0027] Macrobbie, E. (2006) Control of volume and turgor in stomatal guard cells. J. Membr. Biol. 210, 131–142. 16868673 10.1007/s00232-005-0851-7

[pbi12824-bib-0028] Martinoia, E. , Maeshima, M. and Neuhaus, H.E. (2007) Vacuolar transporters and their essential role in plant metabolism. J. Exp. Bot. 58, 83–102. 17110589 10.1093/jxb/erl183

[pbi12824-bib-0029] Mortazavi, A. , Williams, B.A. , Mccue, K. , Schaeffer, L. and Wold, B. (2008) Mapping and quantifying mammalian transcriptomes by RNA‐Seq. Nat. Methods 5, 621–628. 18516045 10.1038/nmeth.1226 PMC13303166

[pbi12824-bib-0030] Mubin, M. , Hussain, M. , Briddon, R.W. and Mansoor, S. (2011) Selection of target sequences as well as sequence identity determine the outcome of RNAi approach for resistance against cotton leaf curl geminivirus complex. Virol. J. 8, 122. 21410988 10.1186/1743-422X-8-122 PMC3315792

[pbi12824-bib-0031] Nieves‐Cordones, M. , Al Shiblawi, F.R. and Sentenac, H. (2016) Roles and transport of sodium and potassium in plants. Met. Ions Life Sci. 16, 291–324. 26860305 10.1007/978-3-319-21756-7_9

[pbi12824-bib-0032] Osakabe, Y. , Arinaga, N. , Umezawa, T. , Katsura, S. , Nagamachi, K. , Tanaka, H. , Ohiraki, H. *et al*. (2013) Osmotic stress responses and plant growth controlled by potassium transporters in *Arabidopsis* . Plant Cell 25, 609–624. 23396830 10.1105/tpc.112.105700 PMC3608781

[pbi12824-bib-0033] Pettigrew, W.T. (2008) Potassium influences on yield and quality production for maize, wheat, soybean and cotton. Physiol. Plant. 133, 670–681. 18331406 10.1111/j.1399-3054.2008.01073.x

[pbi12824-bib-0034] Pratelli, R. , Lacombe, B. , Gaymard, F. , Thibaud, J.B. and Sentenac, H. (2002) A grapevine gene encoding a guard cell K+ channel displays developmental regulation in the grapevine berry. Plant Physiol. 128, 564–577. 11842160 10.1104/pp.010529 PMC148919

[pbi12824-bib-0035] Sato, A. , Sato, Y. , Fukao, Y. , Fujiwara, M. , Umezawa, T. , Shinozaki, K. , Hibi, T. *et al*. (2009) Threonine at position 306 of the KAT1 potassium channel is essential for channel activity and is a target site for ABA‐activated SnRK2/OST1/SnRK2. 6 protein kinase. Biochem. J. 424, 439–448. 19785574 10.1042/BJ20091221

[pbi12824-bib-0036] Sharma, T. , Dreyer, I. and Riedelsberger, J. (2013) The role of K+ channels in uptake and redistribution of potassium in the model plant *Arabidopsis thaliana* . Front Plant Sci. 4, 224. 23818893 10.3389/fpls.2013.00224 PMC3694395

[pbi12824-bib-0037] Shimazaki, K. , Doi, M. , Assmann, S. and Kinoshita, T. (2007) Light regulation of stomatal movement. Annu. Rev. Plant Biol. 58, 219–247. 17209798 10.1146/annurev.arplant.57.032905.105434

[pbi12824-bib-0038] Song, M. , Wang, S. , Chai, L. , Zhang, S. and Shen, Y. (2017) Characterization of an ABA‐induced and K+ channel gene FaKAT1 that regulates strawberry fruit ripening. J. Plant Growth Regul. 36, 312–322.

[pbi12824-bib-0039] Talbott, L.D. and Zeiger, E. (1996) Central roles for potassium and sucrose in guard‐cell osmoregulation. Plant Physiol. 111, 1051–1057. 12226347 10.1104/pp.111.4.1051 PMC160980

[pbi12824-bib-0040] Talbott, L.D. and Zeiger, E. (1998) The role of sucrose in guard cell osmoregulation. J. Exp. Bot. 49, 329–337.

[pbi12824-bib-0041] Tian, L. , Jia, H.F. , Li, C.L. , Fan, P.G. , Xing, Y. and Shen, Y.Y. (2012) Sucrose accumulation during grape berry and strawberry fruit ripening is controlled predominantly by sucrose synthase activity. J. Hortic. Sci. 87, 661–667.

[pbi12824-bib-0042] Voelker, C. , Gomezporras, J.L. , Becker, D. , Hamamoto, S. , Uozumi, N. , Gambale, F. , Mueller‐Roeber, B. *et al*. (2010) Roles of tandem‐pore k^+^ channels in plants ‐ a puzzle still to be solved. Plant Biol. 12, 56–63. 20712621 10.1111/j.1438-8677.2010.00353.x

[pbi12824-bib-0043] Wang, Z.Y. , Fang, B.P. , Chen, J.Y. , Zhang, X.J. , Luo, Z.X. , Huang, L.F. , Chen, X.L. *et al*. (2010) De novo assembly and characterization of root transcriptome using illumina paired‐end sequencing and development of cssr markers in sweet potato (ipomoea batatas). BMC Genet. 11, 726. 10.1186/1471-2164-11-726 PMC3016421 21182800

[pbi12824-bib-0044] Zhang, M. , Yuan, B. and Leng, P. (2009) The role of ABA in triggering ethylene biosynthesis and ripening of tomato fruit. J. Exp. Bot. 60, 1579–1588. 19246595 10.1093/jxb/erp026 PMC2671613

[pbi12824-bib-0045] Zhao, C. , Hua, L.N. , Liu, X.F. , Li, Y.Z. , Shen, Y.Y. and Guo, J.X. (2017) Sucrose synthase *FaSS1*, plays an important role in the regulation of strawberry fruit ripening. Plant Growth Regul. 81, 175–181.

