# Supplementary material for: The potassium channel FaTPK1 plays a critical role in fruit quality formation in strawberry (Fragaria × ananassa)
Source: Plant Biotechnol J. 2017 Oct 12;16(3):737–48. doi: 10.1111/pbi.12824 (PMC5814577; doi:10.1111/pbi.12824)
Supplement: Supplementary file 1 — Figure S1 Nucleotide blast of FaTPK1 gene and FvTPK1 gene. Table S1 Element contents in ripening fruits (FR) and 7‐stage fruits (average) Table S2 The primers used for SqRT‐PCR Table S3 The primers used for qPCR [file PBI-16-737-s001.docx]

**Supplemental Data**

**Figure** **S1.** Nucleotide blast of *FaTPK1* gene and *FvTPK1* gene.


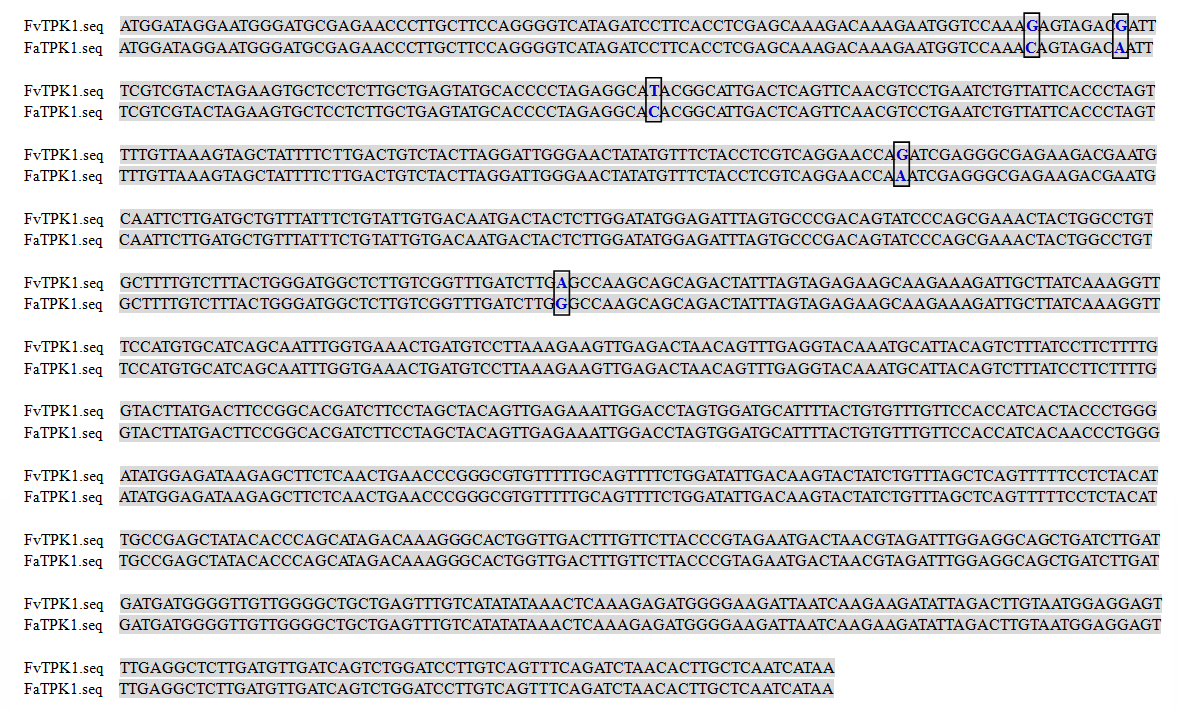


**Table S1.** Element contents in ripening fruits (FR) and 7-stage fruits (average).

| (mg·g-1FW) | K | Ca | Na | Mg | Fe | Mn | Zn | Cu |
| --- | --- | --- | --- | --- | --- | --- | --- | --- |
| FR | 8.283 | 1.179 | 0.221 | 1.351 | 0.129 | 0.022 | 0.017 | 0.004 |
| Average | 8.625 | 1.372 | 0.353 | 1.546 | 0.159 | 0.023 | 0.0152 | 0.004 |

Note: The data was the average value of 3 replicates.

**Table S2.** The primers used for SqRT-PCR.

| Genes | Sequences |
| --- | --- |
| *FaTPK1* | Sense: 5′-TGCTTCCAGGGGTCATAG-3′ |
|  | Antisense: 5′-AGTCTGCTGCTTGGCTCA-3′ |
| *GAL6* | Sense: 5′-GACGGTCAGCATAAGATTC-3′ |
|  | Antisense: 5′-GGCATACCATACTCATCAAG-3′ |
| *PG1* | Sense: 5′- ACGGTTCAGGTTGATGGC -3′ |
|  | Antisense: 5′- GTTCCGATGTTCGTGTCC -3′ |
| *XYL2* | Sense: 5′-GCCTCTATCTACTGTGCGTC-3′ |
|  | Antisense: 5′-TGAATCCCAAACTGGTCTAC-3′ |
| *SUT1* | Sense: 5′- TTCTTGGCTATGCAACTGGA -3′ |
|  | Antisense: 5′- AAACCACCCAATCCAGTTT -3′ |
| *CHS* | Sense: 5′-AGAATCCCAGTATGTGCG-3′ |
|  | Antisense: 5′-TGAGGAGGTGAAATGTGAG-3′ |
| *CHI* | Sense: 5′-CCAGCAATACTCCGAGAA-3′ |
|  | Antisense: 5′-GTCCACTTTCCCATTTCC-3′ |
| *Actin* | Sense: 5′-TGCATATATCAAGCAACTTTACACTGA-3′ |
|  | Antisense: 5′-ATAGCTGAGATGGATCTTCCTGT-3′ |

**Table S3.** The Primers used for qPCR.

| Genes | Sequences |
| --- | --- |
| *FaTPK1* | Sense: 5′-ATGGATAGGAATGGGATGC-3′ |
|  | Antisense: 5′-TGAACTGAGTCAATGCCGTA-3′ |
| *GAL6* | Sense: 5′-CAGGTTGGGTTACTTGGACA-3′ |
|  | Antisense: 5′-TTAGTGGTTTATTGAAAGAGCC-3′ |
| *PG1* | Sense: 5′- GCAAGTAGAGTCGCACAGT -3′ |
|  | Antisense: 5′- TCAGTATTAGGCTTCCCACC -3′ |
| *XYL2* | Sense: 5′-TGTGATAGAGTTGGCAAGATG-3′ |
|  | Antisense: 5′-CGAGAGAAAGAGTGAGGAGG-3′ |
| *SUT1* | Sense: 5′- TTCAAGCGACAGAAATACCC -3′ |
|  | Antisense: 5′- ACCCAATCCAGTTTAGACCAG -3′ |
| *CHS* | Sense: 5′-CATACCCCGACTACTACTTTCGT-3′ |
|  | Antisense: 5′-CGCACATACTGGGATTCTCTT-3′ |
| *CHI* | Sense: 5′-AGCGAAAGCCATTGAAAAGT-3′ |
|  | Antisense: 5′-CATTTGGTGATTGTGTGAAGAG-3′ |
| *Actin* | Sense: 5′-TGCATATATCAAGCAACTTTACACTGA-3′ |
|  | Antisense: 5′-ATAGCTGAGATGGATCTTCCTGT-3′ |
